# The Use of Ion Chromatography for the Determination of Clean-In-Place-200 (CIP-200) Detergent Traces

**Published:** 2007-02-27

**Authors:** Wilfredo Resto, Joan Roque, Rosamil Rey, Héctor Colón, José Zayas

**Affiliations:** 1 Department of Chemistry University of Puerto Rico-Cayey, Antonio R. Barceló Ave. Cayey P.R. 00736; 2 Research and Development Division, ZAYCOR Industries Corporation, 828 de Diego Ave. Caparra Terrace, San Juan PR 00921; 3 Interamerican University, Bayamón Campus, Department of Natural Sciences, Bayamon, Puerto Rico 00957

**Keywords:** cleaning validation, ion chromatography, CIP detergent, phosphate analysis

## Abstract

Anion chromatography with conductivity detection was chosen as the analytical technique for the development of a cleaning validation method for clean-in-place (CIP) detergents. The method was developed and validated for the determination of traces of the detergent CIP-200. It was shown to be linear with a squared correlation coefficient (r^2^) of 0.9999 and the accuracy experiments presented average recoveries of 88.2% (area response factor) from stainless steel surfaces. The repeatability was found to be 1.6% and an intermediate precision of 1.9% across the range. The method was also shown to be sensitive with an average Detection Limit (DL) of 0.23 ppm and a Quantitation Limit (QL) of 0.70 ppm based on the amount of phosphate in the detergent sample. The phosphate signal was well resolved from typical ions encountered in water samples or any other interference presented from swabs and surfaces. The method was applied to cleaning validation samples and proved to be suitable for rapid and reliable quality control.

## Introduction

Ion chromatography (IC) is one of the most employed chromatographic methods nowadays in Industry. Within the different methods included under Ion Chromatography, anion chromatography presents the most popular due to its simplicity, sensitivity and selectivity (Lucy, 1996; Sarzanini, 2001). Anion chromatography can be performed with or without suppression, however, suppressed methods are the most widely used. Reviews on the basic theory and recent advances of IC have been published and the reader is encouraged to read those references for detailed description of the technique as well as the advances related to it (Lucy, 1996; Sarzanini, 2001).

When suppressed methods are employed the eluents commonly used are either carbonate or hydroxide. In the technique, a device known as a suppressor is placed between the column and the detector. With suppression methods, the detection system is based in conductivity. The basis of suppression is the reduction of the background conductivity, while the sensitivity of the anion is increased. The suppression system that takes place in anion chromatography is a cation exchanger that exchanges cations with H^+^ ions. For example, an eluent made of sodium carbonate having a conductivity of ~ 800 μS is converted in the suppression process to carbonic acid which has a conductivity of ~ 18 μS. However, an analyte made of NaCl with a conductivity of ~ 126 μS without suppression, would become HCl with a conductivity of ~ 426 μS when suppression takes place.

For many chromatographers, ion chromatography advances departed with the report by Small in 1975. In this report, Small introduced the use of low capacity stationary phases with suppressed conductivity. (Small, Stevens, and Bauman, 1975) Since then, Anion chromatography has found its niche in the analysis of many substances in many different areas. The first standard method for anion chromatography was established for analysis of anions in water samples in 1984. (ASTM, 1984) Since then, many authors have reported on the use of ion chromatography for the determination of anions.(Jackson and Pohl, 1997; Roig-Navarro, Martínez-Bravo, López et al. 2001; Vanatta and Coleman, 2001.)

When analyzing CIP systems, the characterization of such analytes can be troublesome. The basic reason is that the ratio of components is approximated to percentages ranging from 5 to 20% of each component, as provided by the manufacturer in the MSDS of the cleaning products. Therefore, it is very difficult to attain the right amount for each component. Other methods of analysis include complexation reactions to produce a chromophore suitable for UV detection. This method, is the most common one for the analysis of CIP’s detergents, however, the extra step of the complexation reaction adds the possibility of errors in the analysis. The use of non specific methods such as total organic carbon (TOC) would provide the amount of carbon present in the sample. This amount of carbon however will not necessarily come from the detergent and possible contaminants different than the detergent could be attributed to the presence of the CIP-200. Besides, the exact amount of carbon in the detergent is not known because the amounts of the surfactants used are also reported as a range.

Our laboratory has investigated on ways to analyze CIP detergents for cleaning validation purposes. A healthy cleaning validation program should assure lower levels of cross contamination with products or detergents. The Food and Drug Administration (FDA) enforces those cleaning processes and the Agency published guides where they specified that no detergent should remain after the cleaning process.(FDA, 1993; 2000)

In the present work, we report on the development of a cleaning validation method for the analysis of CIP-200 using the phosphate ion as the analyte to determine traces amounts of the detergent in stainless steel plates. All the parameters required by ICH for the validation of such method were taken into consideration.

## Experimental

### Equipment

The IC system consisted of a Metrohm-peak 761 compact Ion Chromatography system (Herisau, Switzerland) with conductivity detection and a computer with ICNet 2.1 computer software for data handling.

### Materials and reagents

All solvents used were of HPLC and analyticalreagent grade. Water used for mobile phase, sample and standards preparations was obtained from a Barnstead NanoPure (Dubuque, Iowa, U.S.A) system without further purification. The certified ACS sodium bicarbonate was obtained from Fisher (Fair Lawn, NJ, U.S.A), the sodium carbonate was obtained J.T. Baker (Philipsburg, NJ, U.S.A), and the Sodium Phosphate was obtained from Sigma (St. Louis, MO). Stainless steel plates were 25 × 25 mm dimensions, made out of stainless steel 304 un-polished material. The CIP-200 detergent was supplied by the Steris Corporation, lot 216811 (St. Louis, MO, U.S.A).

### Chromatographic conditions

The column used was a Metrohm-Peak Metrosep A Supp 5–150, polyvinyl alcohol with quaternary ammonium groups, 5 μm, and 4.0 mm × 150 mm with a mobile phase composed of sodium carbonate: sodium hydrogen carbonate (3.2 mM: 1.0 mM), flow rate of 0.7 mL/min. The injection volume used was 20 μL. The chromatographic experiments were run at room temperature (20 °C).

### Mobile phase preparation

The sodium carbonate: sodium hydrogen carbonate (4.5 mM: 1.0 mM) mobile phase was prepared by weighing 0.48 g of sodium carbonate and 0.097 g of sodium hydrogen carbonate dissolved them with deionized water and transferred to a 1.00 L volumetric flask and diluted to volume with deionized water. The mixture was properly filtered and degassed. This solution was used as the mobile phase, diluent for the phosphate standards and CIP-200 working samples, and also as the extracting solution.

### Preparation of the phosphate standards

The phosphate stock standard solution was prepared by weighing 0.0971 g Na_3_PO_4_ dissolved in deionized water and transferred to a 100.00 mL volumetric flask and diluted to volume with deionized water. The phosphate working solution was prepared by pipetting 5.00 mL of the stock solution into a 50.00 mL volumetric flask and diluted to volume with mobile phase. The resulting concentration for the phosphate anion in the stock standard solution and the working solutions were 562 ppm and 56.2 ppm respectively. From the working solution different aliquots were taken and diluted to volume with the sodium carbonate: sodium hydrogen carbonate (4.5 mM: 1.0 mM) mobile phase. Three replicates were prepared for each off the standards solutions. The final concentrations of the standards solutions are presented in Table 1.

### Preparation of stock CIP-200 detergent solutions

A 0.25 mL aliquot of a CIP-200 sample was placed in a 100.00 mL volumetric flask and diluted to volume with deionized water. Aliquots of this stock solution were further diluted in order to reach the desired concentration for these studies.

### Preparation of the CIP-200 sample

The sample for the determination of phosphate concentration in the CIP-200 was prepared by pipetting 0.50 mL of the CIP-200 stock solution to a 10.00 mL volumetric flask and diluted to volume with the sodium carbonate: sodium hydrogen carbonate (4.5 mM: 1.0 mM) mobile phase. From this solution 1.00 mL were pipetted into a 10.00 mL volumetric flask and diluted to volume with mobile phase. Three replicates were prepared for the CIP sample. The average phosphate concentration determined for this solution was 6.74 ppm by area response factor and 6.77 ppm by height response factor.

### Preparation for the recovery of CIP-200 from stainless steel surface

The solutions used for recovery from plate were prepared using aliquots from the CIP-200 stock solution. Aliquots of 2.00 mL, 2.50 mL, and 3.00 mL were pipetted to 25.00 mL volumetric flasks and diluted to volume with the sodium carbonate: sodium hydrogen carbonate (4.5 mM: 1.0 mM) mobile phase. From the determination of the CIP-200 sample the resulting concentrations for these solutions were 108 ppm, 135 ppm, and 162 ppm respectively. A volume of 100 μL for each of these solutions was spread over a clean and dry 2″ × 2″ stainless steel plate. The procedure was repeated for each of the solutions. The metal plates were allowed to dry at room temperature. A TEXWIPE TX761 swab was deposited in a vial that contained 2.00 mL of mobile phase. For each deposited aliquot, a wet swab was passed over the surface of the plate, one side of the swab was passed horizontally and the other vertically. The swabbing process has been represented schematically elsewhere.(Zayas, Colón, Garced, et al. 2006) The swab was returned to a vial with 2.00 mL of mobile phase. The vials were shaken mechanically for 10 minutes and each of them analyzed by IC.

## Results and Discussion

### System suitability

**Figure 1.**
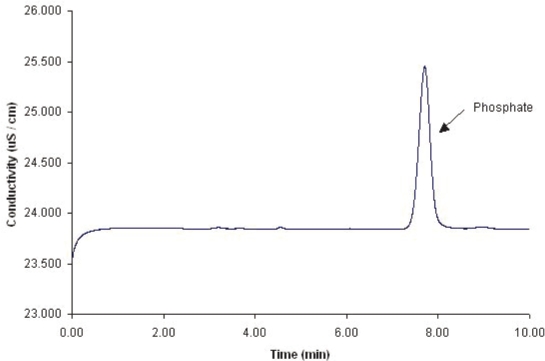
Typical system suitability chromatogram of the Phosphate Standard. Suitability ran at room temperature at 0.7 mL/min. Na_2_CO_3_-NaHCO_3_: 4.5 mM: 1.0 mM. Conductivity detection.

**Figure 2.**
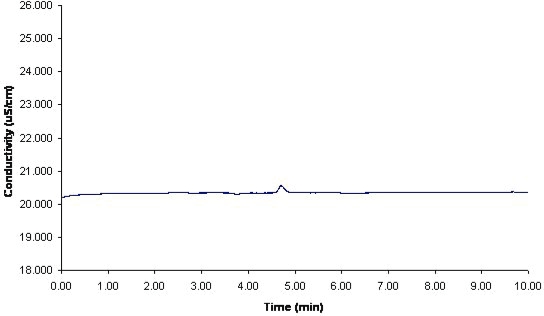
Typical blank chromatogram. Ran at room temperature at 0.7 mL/min. Na_2_CO_3_-NaHCO_3_: 4.5 mM: 1.0 mM. Conductivity detection.

The ion chromatographic system suitability was evaluated according to the requirements set forth by the United States Pharmacopoeia (USP 27). (21CFR 211.67; 21CFR 211.160, United States Pharmacopeia (USP) 2004) System precision, theoretical plates (N), and tailing factor (T) were evaluated. The system precision was obtained from the pooled relative standard deviation (co-variance, percentage of RSD_pooled_) of three sets of replicate injections from different days and preparations. Each replicate set consisted of six consecutive injections. This afforded a percentage of RSD_pooled_ value of 0.51% by area response factor and 1.1% by height response factor. The average theoretical plates resulted in N of 3900, and the tailing factor, T, was calculated at 1.1 on the average. The resolution factor R was calculated against the chlorine peak and set at 6 based on average determinations. Figure 1 displays a typical chromatogram for a system suitability run. Figure 2 displays a typical blank chromatogram.

### Repeatability and intermediate precision

The repeatability of the method was determined by using the response factor values obtained for a set of different concentrations. The set consisted of three consecutive injections for each of the three different concentrations. These were averaged and the pooled standard deviation determined (S_pooled_). These values were used to calculate the pooled percentage of RSD. This afforded a percentage of RSD_pooled_ value of 1.1% by area response factor and 1.6% by height response factor.

The intermediate precision of the method was determined by using the response factor values for a set of different concentrations prepared by different analysts on the same day and by the same analyst on different days. Each set consisted of three consecutive injections for each of the three different concentrations. These were averaged and the pooled standard deviation determined (S_pooled_). The values were used to calculate the pooled percentage of RSD. This afforded a percentage of RSD_pooled_ value of 1.9% by area response factor and 2.1% by height response factor. Table 2 shows the pooled chromatographic data used for the calculations.

### Ruggedness

The ruggedness was demonstrated by comparing the recovery from the plate for two different preparations by two different analysts. The statistical calculations are explained elsewhere. (Zayas, Colón, Garced et al. 2006) From the results it can be concluded that the method is rugged enough to allow two different analysts to work on the determination of phosphate in CIP-200 without significant statistical differences.

### Linearity

**Figure 3.**
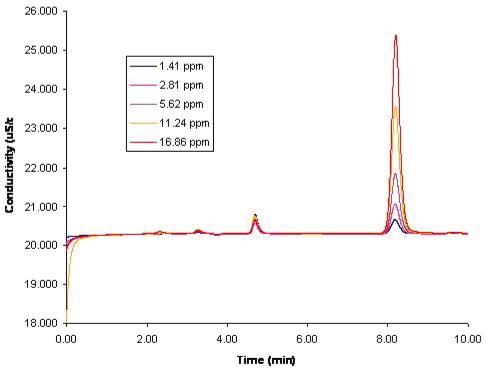
Typical calibration chromatograms of the phosphate standards. Ran at room temperature at 0.7 mL/min. Na_2_CO_3_-NaHCO_3_: 4.5 mM: 1.0 mM. Conductivity detection.

The linearity of the method was established by calculating the linear regression of multiple determinations at a concentration range from 1.41 ppm to 16.83 ppm of phosphate standards. The data was combined to determine the linearity of the method. The calibration curve showed a sensitivity of 4.49 (μS/cms)/ppm with correlation coefficient of 0.9999 for the area response factor and sensitivity of 0.308 (μS/cm)/ppm with a correlation coefficient of 0.9993 for the height response factor. The method demonstrated outstanding linearity over the concentration range analyzed. The data of the calibration curve of phosphate standards and the CIP-200 phosphate determination is shown in Table 3. Figure 3 presents a stacked arrangement of the typical chromatograms of the phosphate standards.

### Limit tests

The detection limit (DL) and the quantitation limit (QL) were determined from the calibration curve of 5 different phosphate standard concentrations. The ICH guide (ICH, 1996) recommends as an alternative for the estimation of the Detection (DL) and Quantitation (QL) limits the following equation:

S/N Estimate =Sxy/Slope

Where S/N Estimate is the approximation of the signal-to-noise ratio (semi-empirical), Sxy is the standard error of the intercept, and the slope of the linear regression curve from the Linearity determination. Multiplying the S/N Estimate by 3.3 and 10 affords the estimate of the DL and QL, respectively. This calculation yielded an average DL of 0.23 ppm, and an average QL of 0.70 ppm, both of them by area response factor. These calculations were based on the amount of phosphate present in CIP-200. In order to correlate this number to the amount of CIP-200 present, the amount of phosphate contained in a sample of CIP-200 must be determined. Then, taking into consideration the calculated content of phosphate in CIP-200, the DL for CIP would be around 0.11 nL and the quantitation limit for CIP-200 would be around 0.32 nL of the detergent.

### Determination of phosphate in CIP-200 detergent/accuracy experiments

**Figure 4.**
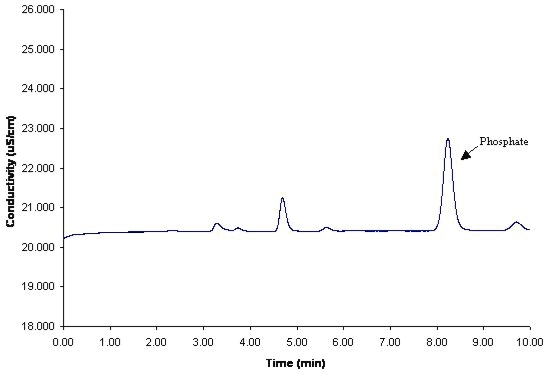
Typical CIP-200 chromatogram for the accuracy experiments. Chromatogram ran at room temperature at 0.7 mL/min. Na_2_CO_3_-NaHCO_3_: 4.5 mM: 1.0 mM. Conductivity detection.

A set of recovery experiments were performed to assess the accuracy and precision of the method using CIP-200 samples. A concentration range going from 1.41 ppm to 16.9 ppm of phosphate was used as the calibration curve. This estimated recovery was obtained by dividing the response factor of each concentration recovered and divided by the slope of the linear regression curve of the found versus theoretical concentration for the phosphate. The CIP-200 samples were extracted form the cotton swab using mobile phase as the extracting solvent. 100 μL of diluted CIP-200 deposited on the stainless steel plates was diluted further in 2.00 mL of mobile phase and after that 20 μL of that was injected into the ion chromatographic system. The average % recovery of the CIP-200 samples was calculated to be 90.1% for the area response factor, and 90.9 % for the height response factor. Table 4 and Table 5 presents the data obtained from a typical accuracy experiment of CIP-100 from the stainless steel plates for area response factor and the height response factor, respectively. Figure 4 shows a typical chromatogram of the ion chromatography analysis of phosphate contained in a CIP-200 detergent sample.

## Conclusions

Anion Exchange chromatography proved to be an excellent analytical technique for the determination of CIP-200 traces. The developed anion exchange chromatographic method has been evaluated over the linearity, precision, accuracy, and selectivity and proved to be convenient and effective for the quality control of cleaning validation samples. The method is fast and reliable affording turn around times convenient for the quality control laboratory. Solvents are mostly aqueous and its consumption is low which makes the method environmentally friendly. The DL and QL of the method are less than 1 ppm of phosphate in the CIP-200 sample which translates to less than 1 nL of CIP-200, making it an excellent method for the determination of traces of CIP-200 in cleaning validation.

## Figures and Tables

**Table 1.** Phosphate standard preparation (10.00 mL final volume)

**Aliquot of phosphate working solution (mL)**	**Theoretical concentration of phosphate (ppm)**
0.25	1.41
0.50	2.81
1.00	5.62
2.00	11.2
3.00	16.9

**Table 2.** Pooled Chromatographic Data to Asses Intermediate Precision.

**Standard Concentration (ppm)**	**Average Area (μS/cms)**	**Pooled Standard Deviation (μS/cms)**	**Pooled RSD%**	**Average Height (μS/cm)**	**Pooled Standard Deviation (μS/cm)**	**Pooled RSD%**
2.81	14.225	0.53	3.76	0.799	0.12	15.13
5.62	29.329	0.49	1.68	1.660	0.03	1.81
11.23	60.036	1.03	1.71	3.422	0.10	2.97

**Table 3.** Calibration Curve and CIP-200 Phosphate Chromatographic Determination Data.

**Standard Concentration (ppm)**	**Average Area (μS/cms)**	**Standard Deviation (μS/cms)**	**RSD%**	**Average Height (μS/cm)**	**Standard Deviation (μS/cm)**	**RSD%**
1.41	5.596	0.06	1.12	0.363	0.01	1.59
2.81	11.786	0.24	2.06	0.770	0.01	1.30
5.62	24.083	0.13	0.53	1.567	0.01	0.37
11.2	49.133	0.41	0.84	3.287	0.02	0.46
16.9	74.994	0.47	0.63	5.127	0.04	0.69
CIP-200 Sample	29.333	0.20	0.67	1.97	0.01	0.72

**Table 4.** Typical Accuracy Experiment Data for Area Response Factor.

**Deposited Concentration (ppm)**	**Expected Concentration (ppm)**	**Average Area (μS/cms)**	**Calculated Concentration (ppm)**	**% Recovered**
108	5.40	19.407	4.53	84.0
135	6.75	26.918	6.20	92.0
162	8.10	33.337	7.63	94.3

**Table 5.** Typical Accuracy Experiment Data for Height Response Factor.

**Deposited Concentration (ppm)**	**Expected Concentration (ppm)**	**Average Height (μS/cm)**	**Calculated Concentration (ppm)**	**% Recovered**
108	5.40	1.30	4.59	85.0
135	6.75	1.82	6.28	93.0
162	8.10	2.25	6.82	94.8
